# P-245. Dolutegravir Use over 48 Weeks is Not Associated With Worsening Insulin Resistance and Pancreatic Beta Cell Function in a Cohort of HIV-infected Ugandan Adults

**DOI:** 10.1093/ofid/ofaf695.467

**Published:** 2026-01-11

**Authors:** Frank Mulindwa, Barbara Castelnuovo, Jean-Marc Schwarz, Robert C Bollinger, Nele Brusselaers

**Affiliations:** United Health Services, Wilson Hospital, Johnson City, NY; Makerere University Infectious Diseases Institute, Kampala, Kampala, Uganda; University of California San Francisco, San Francisco, California; Johns Hopkins , MD; University of Antwerp, Antwerp, Antwerpen, Belgium

## Abstract

**Background:**

There have been case reports and series of persons with HIV (PWH) developing hyperglycemia within weeks to months on starting integrase inhibitors. At population level however with longer follow up, meta-analyses have demonstrated a reduced risk of incident diabetes mellitus with longer follow up as compared to protease inhibitors and non-nucleoside reverse transcriptase inhibitors. We sought to demonstrate changes in insulin resistance and pancreatic beta cell function in PWH in the first 48 weeks on Dolutegravir/ lamivudine/ Tenofovir Disoproxil Fumarate to demonstrate if there is an acute transient worsening of insulin resistance/ pancreatic beta cell function in the first weeks of dolutegravir use.

**Table 1:**
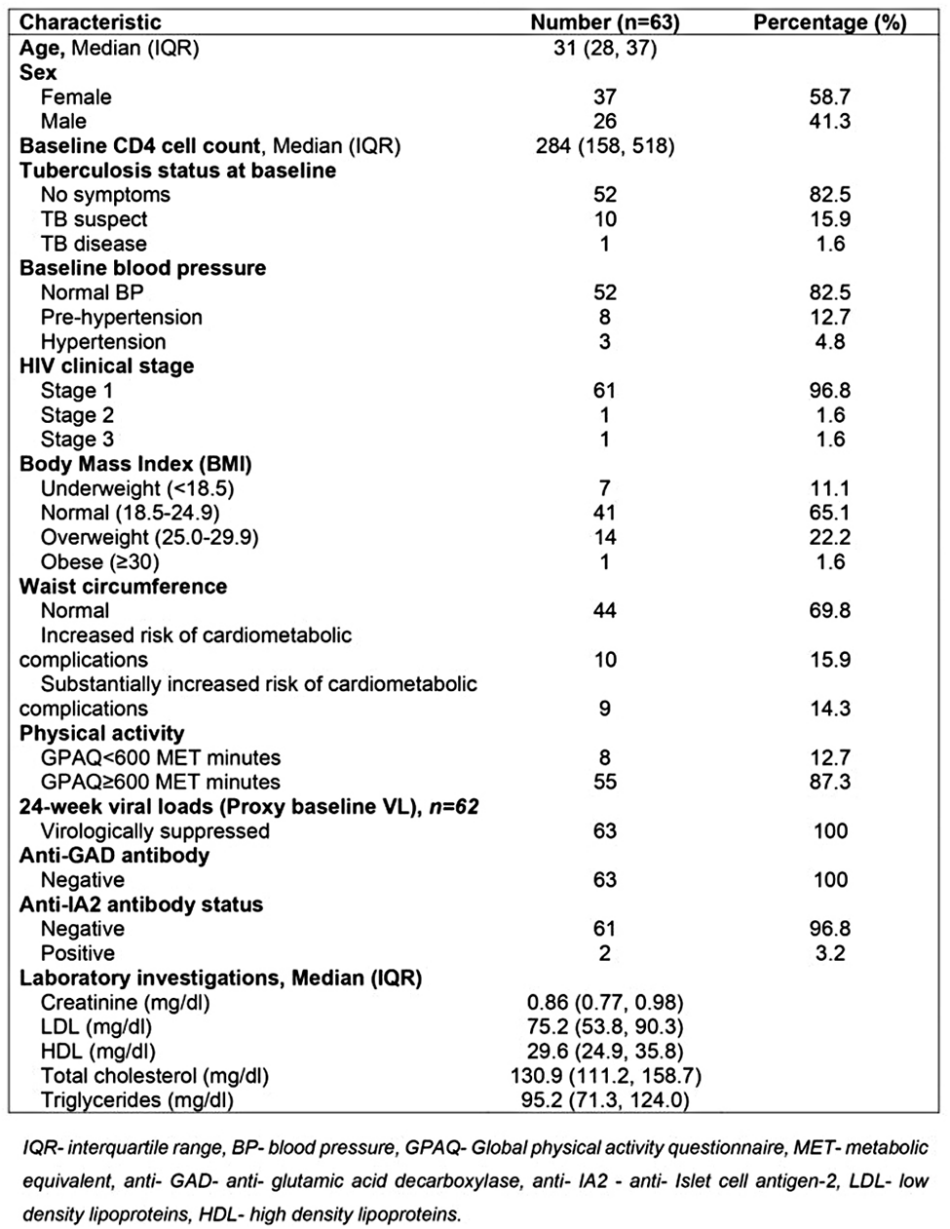
Baseline clinical and demographic characteristics of the study participants.

**Table 2.**
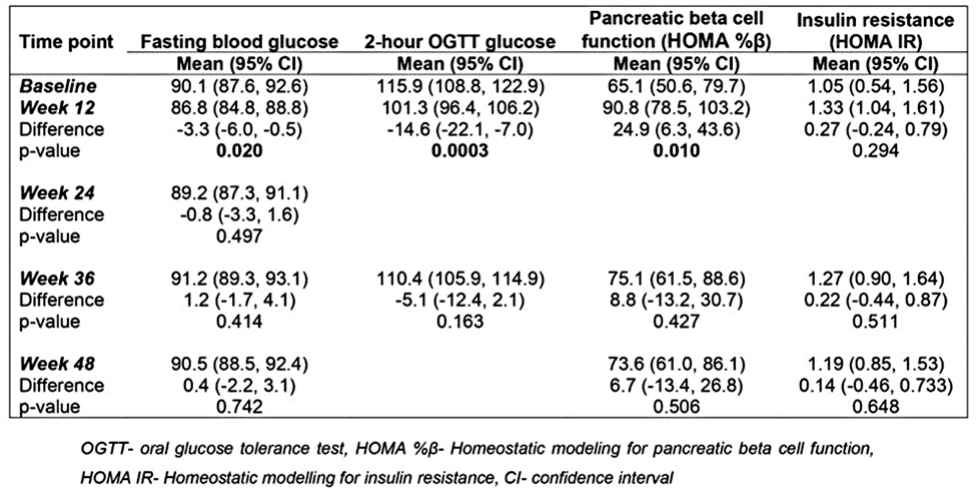
Changes in blood glucose, pancreatic beta cell function and insulin resistance over 48 weeks on dolutegravir in the study participants

**Methods:**

In this analysis, 63 patients underwent serial oral glucose tolerance tests over 48 weeks. Using fasting serum insulin and glucose, we calculated insulin resistance and pancreatic beta cell function by homeostatic modelling (HOMA IR and HOMA%β respectively). Absolute mean changes between baseline and post-baseline blood glucose, pancreatic beta cell function and insulin resistance were computed by subtracting each post-baseline value from the baseline value and compared using student t-test.

**Figure 1:**
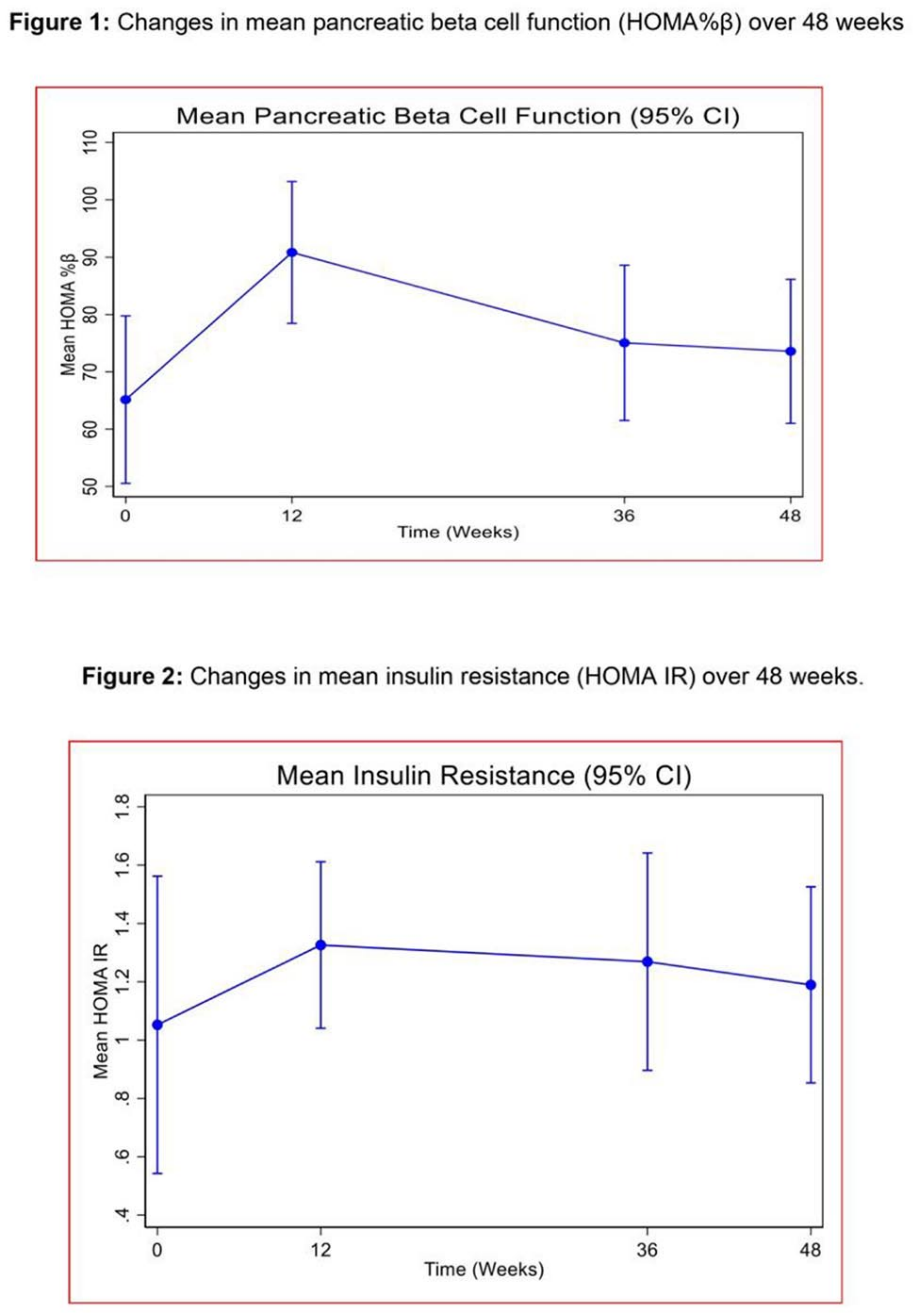
Changes in mean pancreatic beta cell function (HOMA%β) and insulin resistance (HOMA IR) over 48 weeks

**Results:**

Of the 63 participants, 37 (58%) were female. Median age was 31 (IQR: 28-37). Despite a trend towards an initial increase in both HOMA IR and HOMA%β at 12 weeks followed by a decline through 36 weeks to 48 weeks, the HOMA IR and HOMA%β at 48 weeks were not significantly different from baseline i.e. (difference in mean HOMA IR from baseline: 0.14, 95%CI: -0.46, 0.733, p= 0.648) and (difference in mean HOMA %β from baseline: 6.7, 95%CI: -13.4, 26.8, p= 0.506) respectively.

**Conclusion:**

We demonstrated that both insulin resistance and pancreatic beta cell function were unchanged among Ugandan PWH on dolutegravir over 48 weeks. We add to the body of evidence demonstrating glucose metabolic safety of dolutegravir in ART naïve patients.

**Disclosures:**

Robert C. Bollinger, Jr., MD, MPH, [SCENE] Health: Advisor/Consultant|[SCENE] Health: Board Member|[SCENE] Health: Stocks/Bonds (Private Company)|Merck: Advisor/Consultant|miDiagnostics: Co-inventor of IP owned by Johns Hopkins University|miDiagnostics: Eligible for equity and royalty payments received by Johns Hopkins University

